# Advances in Host Depletion and Pathogen Enrichment Methods for Rapid Sequencing–Based Diagnosis of Bloodstream Infection

**DOI:** 10.1016/j.jmoldx.2024.05.008

**Published:** 2024-06-24

**Authors:** Mohammad S. Islam Sajib, Kirstyn Brunker, Katarina Oravcova, Paul Everest, Michael E. Murphy, Taya Forde

**Affiliations:** ∗ School of Biodiversity, One Health and Veterinary Medicine, University of Glasgow, Glasgow, United Kingdom; † Medical Research Council–University of Glasgow Centre for Virus Research, Glasgow, United Kingdom; ‡ Department of Microbiology, National Health Service Greater Glasgow and Clyde, Glasgow, United Kingdom; § School of Medicine, Dentistry and Nursing, University of Glasgow, Glasgow, United Kingdom

## Abstract

Bloodstream infection is a major cause of morbidity and death worldwide. Timely and appropriate treatment can reduce mortality among critically ill patients. Current diagnostic methods are too slow to inform precise antibiotic choice, leading to the prescription of empirical antibiotics, which may fail to cover the resistance profile of the pathogen, risking poor patient outcomes. Additionally, overuse of broad-spectrum antibiotics may lead to more resistant organisms, putting further pressure on the dwindling pipeline of antibiotics, and risk transmission of these resistant organisms in the health care environment. Therefore, rapid diagnostics are urgently required to better inform antibiotic choice early in the course of treatment. Sequencing offers great promise in reducing time to microbiological diagnosis; however, the amount of host DNA compared with the pathogen in patient samples presents a significant obstacle. Various host-depletion and bacterial-enrichment strategies have been used in samples, such as saliva, urine, or tissue. However, these methods have yet to be collectively integrated and/or extensively explored for rapid bloodstream infection diagnosis. Although most of these workflows possess individual strengths, their lack of analytical/clinical sensitivity and/or comprehensiveness demands additional improvements or synergistic application. This review provides a distinctive classification system for various methods based on their working principles to guide future research, and discusses their strengths and limitations and explores potential avenues for improvement to assist the reader in workflow selection.

Bloodstream infection (BSI) is a major public health concern and leads to approximately 2.9 million deaths worldwide each year.^1^ Timely identification of the pathogen and its resistance profile is crucial to inform patient management and improve outcomes. According to some estimates, up to 80% survival rate could be achieved if effective antimicrobials are administered to critically ill patients within 1 hour of developing hypotension compared with ≤42% if treatment is delayed for up to 6 hours.^2^ Traditional culture-based methods unfortunately fail to live up to these criteria because of long turnaround time (48 to 72 hours) to identify pathogen and antimicrobial resistance (AMR) profile.^3^ Consequently, biomarker-based assays combined with improved management guidelines are providing a rapid means of ruling in/out infection and deciding on empirical regimens.^4^ Although strict adherence to these guidelines can lead to reduced mortality,^5^ there remains a pressing need for rapid diagnostic tools to identify the pathogens, AMR, and direct targeted antibiotic therapy. Various nucleic acid amplification technologies, such as PCR, have been used to address this issue. However, overall lack of breadth makes them less ideal as an all-purpose species and/or AMR detection tool.^6^ Therefore, their capacity to guide rapid and appropriate treatment decisions is limited.

Next-generation sequencing (NGS) technologies have revolutionized medical research by allowing us to obtain high-throughput sequencing data for infectious disease diagnosis. Unlike PCR, certain applications of NGS [eg, metagenomic NGS (mNGS)] can identify a wide variety of microorganisms and AMR determinants without the need for a predefined assumption of what is present in the sample (ie, they are pathogen agnostic).^7^ However, sample processing, library preparation, and sequencing can be lengthy and, therefore, obtaining desirable results using NGS within a clinically relevant time frame is challenging.^7^

Advances in sequencing technologies are offering means to overcome some of these obstacles. Oxford Nanopore Technologies (ONT; Oxford, UK; eg, MinION), paired with rapid protocols where DNA libraries can be prepared within 10 minutes (eg, ONT Rapid Barcoding Kit) allow sequencing in real time, whereby results are available immediately for analysis.^8^ Although generally less accurate compared with other platforms (eg, Illumina, San Diego, CA),^9^ ONT has demonstrated gold standard diagnostic accuracy for severe acute respiratory syndrome coronavirus 2 (SARS-CoV-2), and the latest chemistry can achieve Q30 (>99.9%) raw read accuracy.^10^ Competitors like Illumina, Pacific Biosciences (Menlo Park, CA), and Thermo Fisher Scientific (Waltham, MA) also offer rapid sequencing options to prepare a library in <2 hours [eg, Illumina DNA PCR-Free prep kit and NEBNext Fast DNA Library Prep kit for Ion Torrent (New England Biolabs, Ipswich, MA)]. Though data analysis with these platforms can be rapid, it is possible only after completing the sequencing run, which takes approximately 3 to 4 hours with one of their fastest solutions (eg, Illumina MiSeq Reagent Kit V2, Thermo Fisher Scientific Ion 300 series Chip V2, or Pacific Biosciences SMRT RS II).^11^^,^^12^ This offers users the flexibility to select sequencing platforms and/or workflows that best suit their needs.

Despite the availability of these rapid platforms/workflows, one of the biggest challenges for any sequencing-based approaches lies with the sample itself. Blood represents a particularly challenging sample, where the number of white blood cells (cells containing DNA) typically ranges between 4000/μL and 11,000/μL of blood (4 × 10^6^ to 1.1 × 10^7^ cells/mL).^13^ This number can increase (leukocytosis; white blood cell count >12,000) or decrease (leukopenia; white blood cell count <4000), depending on the immune status of the patient and the type of pathogen involved.^14^ In comparison, bacterial load is mostly between 1 and 100 colony-forming units (CFUs)/mL, constituting <0.002% of the total nucleated cells in whole blood.^15^ Moreover, the human genome is approximately 3055 million bases^16^ compared with only 3.45 (±1.8) million bases on average (approximately 885× smaller) for bacterial species.^17^ Therefore, even in a one-to-one genome content comparison, total nucleic acid of a bacterium corresponds to only a fraction (approximately 0.1%) of a single mammalian host cell containing DNA. This is a common problem in all samples with a high level of host background (eg, tissue, swabs, and body fluids), and low initial bacterial load.

Considering this, several studies have used high-throughput mNGS on body fluids or cell-free circulating DNA with the aim of obtaining a sufficient number of reads from the desired bacterial species.^18^^,^^19^ Although some of them have suggested that mNGS is equally or more sensitive to culture-based methods,^18^ with only mNGS, the reads classified as nonhost are typically sufficient for predicting the species causing infection but not AMR determinants.^18^^,^^20^ Consequently, acquisition of sufficient mNGS AMR data to inform clinical management results in a substantial amount of time, and increased sequencing cost per sample.^18^^,^^21^

Selective depletion of host and/or enrichment of the pathogen can help improve the host to pathogen ratio, however, only a few depletion/enrichment methods have been tested for diagnosing BSI and/or developed initially as a sequencing workflow. Among them, some techniques are traditional and rely mainly on the physical properties of the host/bacteria (size, shape, and density), categorized as physical methods. More recent approaches try combining both physical and chemical methods (physiochemical) to use their synergistic strengths. In addition, some physiochemical methods are developed only to remove host (depletion) or target and enrich pathogens (enrichment), both of which can be done before or after DNA extraction, allowing the users to choose/combine the most suitable workflow for their application. This review categorizes these methods based on their working principle and discusses their possible applications, advantages, and disadvantages if used for the rapid sequencing-based detection of pathogen and/or AMR determinants directly from blood samples (Figure 1^6^^,^^22^, ^23^, ^24^, ^25^, ^26^, ^27^, ^28^, ^29^, ^30^, ^31^, ^32^, ^33^, ^34^, ^35^, ^36^, ^37^, ^38^, ^39^, ^40^, ^41^, ^42^, ^43^, ^44^, ^45^, ^46^, ^47^, ^48^, ^49^, ^50^, ^51^, ^52^, ^53^, ^54^, ^55^, ^56^). Although some of the primary studies used these techniques on samples not directly associated with BSI (eg, other clinical syndromes), their use remains relevant for infection in general. Therefore, these approaches can be tailored to suit various syndromes/sample types.

**Figure 1:**
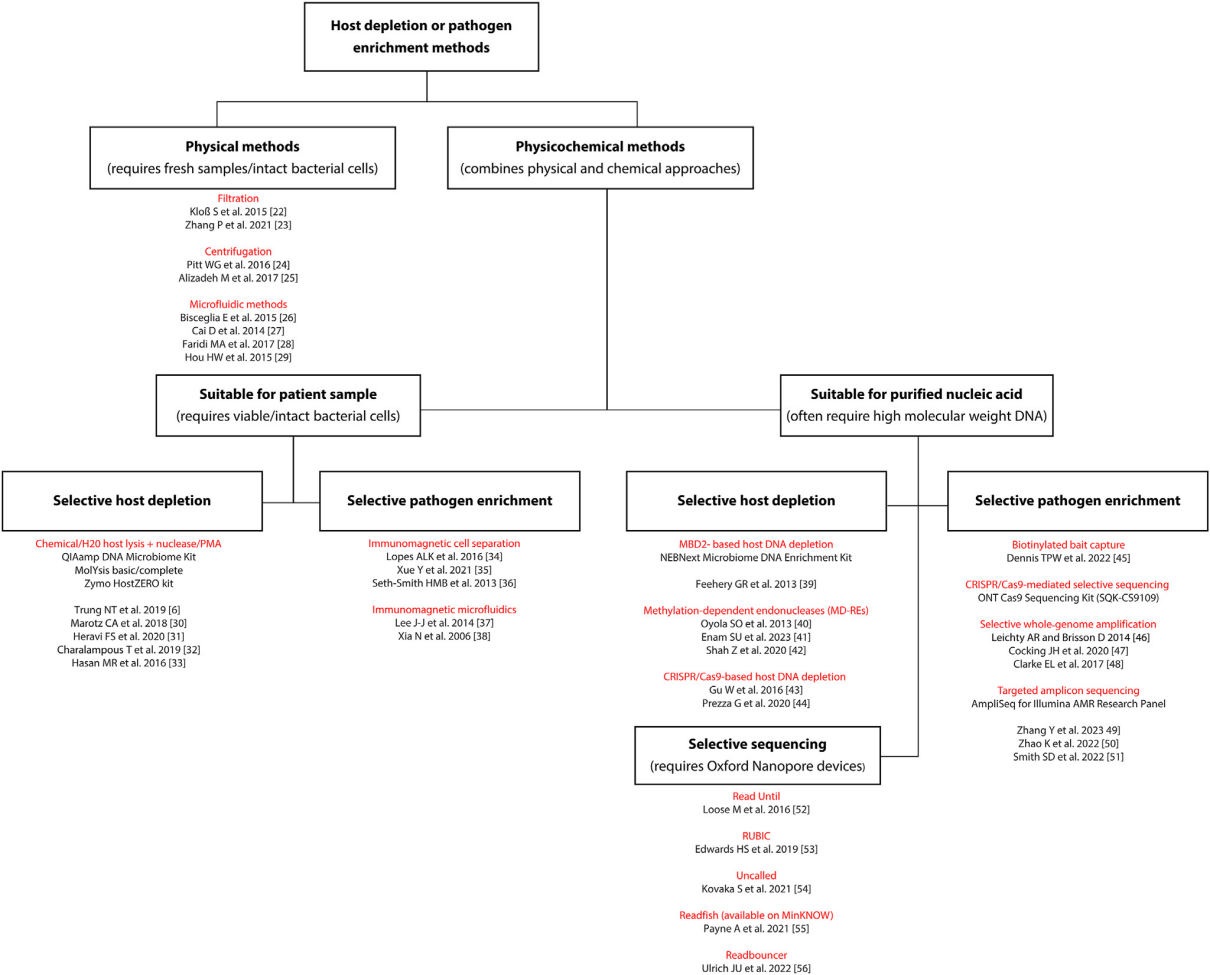
A flowchart showing categories and subcategories of major physical and physicochemical methods for host DNA depletion and/or pathogen DNA enrichment described in the literature and reviewed here, with specific groupings shown in red text. MBD2, methyl-CpG–binding domain protein 2; MD-RE, methylation-dependent restriction endonuclease.

## Physical Methods

Physical separation methods refer to processes wherein mechanical forces are applied to separate and/or concentrate bacteria from a biological sample. Certain chemical compounds might also be used in this approach but only to aid or accelerate the physical separation process (Figure 1 and Supplemental Table S1^6^^,^^22^, ^23^, ^24^, ^25^, ^26^, ^27^, ^28^, ^29^, ^30^, ^31^, ^32^, ^33^, ^34^, ^35^, ^36^, ^37^, ^38^, ^39^, ^40^, ^41^, ^42^, ^43^, ^44^, ^45^, ^46^, ^47^, ^48^, ^49^, ^50^, ^51^, ^52^, ^53^, ^54^, ^55^, ^56^, ^57^, ^58^).

### Filtration

Mechanical filtration takes advantage of the size difference between the bacterial cells and other undesired particles, such as nucleated host cells, in a sample. This process typically involves passing a sample through a membrane with a defined pore size that will selectively allow or restrict bacterial cells to pass, while having the opposite effect on the undesired particles. Physical filtration is relatively simple and rapid and, therefore, numerous studies have used this approach to enrich pathogens from saliva, urine, and cerebrospinal fluid samples.^22^^,^^23^ However, with blood samples having a low concentration of bacteria, efficient separation can be difficult because of the tendency for red blood cells to pack together and form a nonporous layer on top of the membrane.^24^ This obstruction can impede the membrane, hindering a significant proportion of the desired, already low-abundance bacterial cells from passing through. Consequently, this reduces the likelihood of detecting them downstream. It is important to consider that the filtration methods alone might also remove intracellular pathogens along with the host cells they infect and live inside.

### Centrifugation

Differential centrifugation is a technique that uses a rotational force for a certain amount of time (varies by study) to isolate bacterial cells in a sample. This may be used in combination with specialized apparatus (eg, disks, filters, or cartridges) and can include a density gradient medium, such as Ficoll (Sigma-Aldrich, St. Louis, MO), Percoll (Sigma-Aldrich), or OptiPrep (STEMCELL Technologies, Vancouver, BC, Canada).^24^^,^^25^^,^^59^ Although mostly used to fractionate blood, some mNGS-based studies have used centrifugation to deplete host cells by separating plasma from the whole blood samples (eg, cell-free circulating DNA sequencing).^18^ These approaches are rapid and simple; however, host removal and/or recovery of bacterial cells are suboptimal for low abundant samples, such as blood.^25^^,^^60^ The range of bacterial cell size/density often overlaps with some other blood components (eg, platelets or red blood cells), making the removal of host cells less efficient.^24^ Also, host cell-free circulating DNA in plasma (used for mNGS-based BSI diagnosis) or DNA released from nucleated host cells because of improper handling and storage would be impossible to remove using centrifugation alone. This can significantly decrease sequencing yield of the pathogen and, therefore, affect the analytical sensitivity and turnaround time of the sequencing-based assays.

## Microfluidic Methods

Microfluidic approaches with a variety of underlying principles have been described in the literature. Acoustophoresis is one such approach, where sound waves are used to allow selective migration of bacterial cells toward a collection chamber. To date, a few proof-of-principle studies have used acoustophoresis on spiked or clinical blood samples.^61^, ^62^, ^63^ However, their performance has not reached optimal levels, likely because of reduced analytical sensitivity compared with culture (<50% sensitive; limit of detection, 1000 CFUs/mL).^63^ Dielectrophoresis (ie, using a gradient of electric field) is another popular microfluidics technique for bacterial enrichment. Several studies have tested this technique on spiked blood samples with high initial bacterial concentrations (between 10^3^ and 10^4^ CFUs) and using various bacterial species (eg, *Escherichia coli* and *Staphylococcus aureus*), with its efficiency to capture bacterial cells ranging from 30% to 97%.^26^^,^^27^ Bacterial cells have also been enriched from blood or other samples using inertial microfluidics, a technique that relies on forces such as fluid drag, lift, and interactions of particles inside the wall of a microfluidic device.^64^ With appropriate adjustments, these forces can enrich bacteria from samples with varying efficiency (10% to 90%), depending on the bacterial species, bacterial concentration, and composition of the sample.^28^^,^^29^ In 2015, Hou et al^29^ tested an in-house spiral microchannel fluidic device that recovered >65% of bacterial cells from only 10 to 50 CFUs initially spiked in the blood sample. Although the downstream molecular assay showed high analytical sensitivity for identifying bacteria, detecting AMR genes required >10^5^ CFUs/mL bacteria and, therefore, an additional blood culture step following enrichment was recommended.^29^

The efficiency of microfluidic methods reduces significantly with sample complexity. Therefore, most of the protocols described in the literature dilute samples, such as blood, before microfluidic processing steps, which considerably limits their throughput. For sequencing-based diagnosis, microfluidics as a standalone method may also have limitations, like filtration and/or centrifugation, as mentioned in the preceding sections (Supplemental Table S1). However, their utility for rapid BSI diagnosis can be evaluated in combination with other methods, specifically for sequencing-based downstream assays.

## Physicochemical Approaches

Physiochemical methods combine both physical- and chemical-based approaches to harness their collective strengths. A group of these approaches works directly on patient samples (before extraction) and, thus, requires intact host/bacterial cells. Others primarily target nucleic acid (after extraction) to selectively remove host or enrich bacterial genomic material (Figure 1 and Supplemental Table S1).

## Physiochemical Methods Suitable for Patient Samples

### Host Depletion

**Figure 2:**
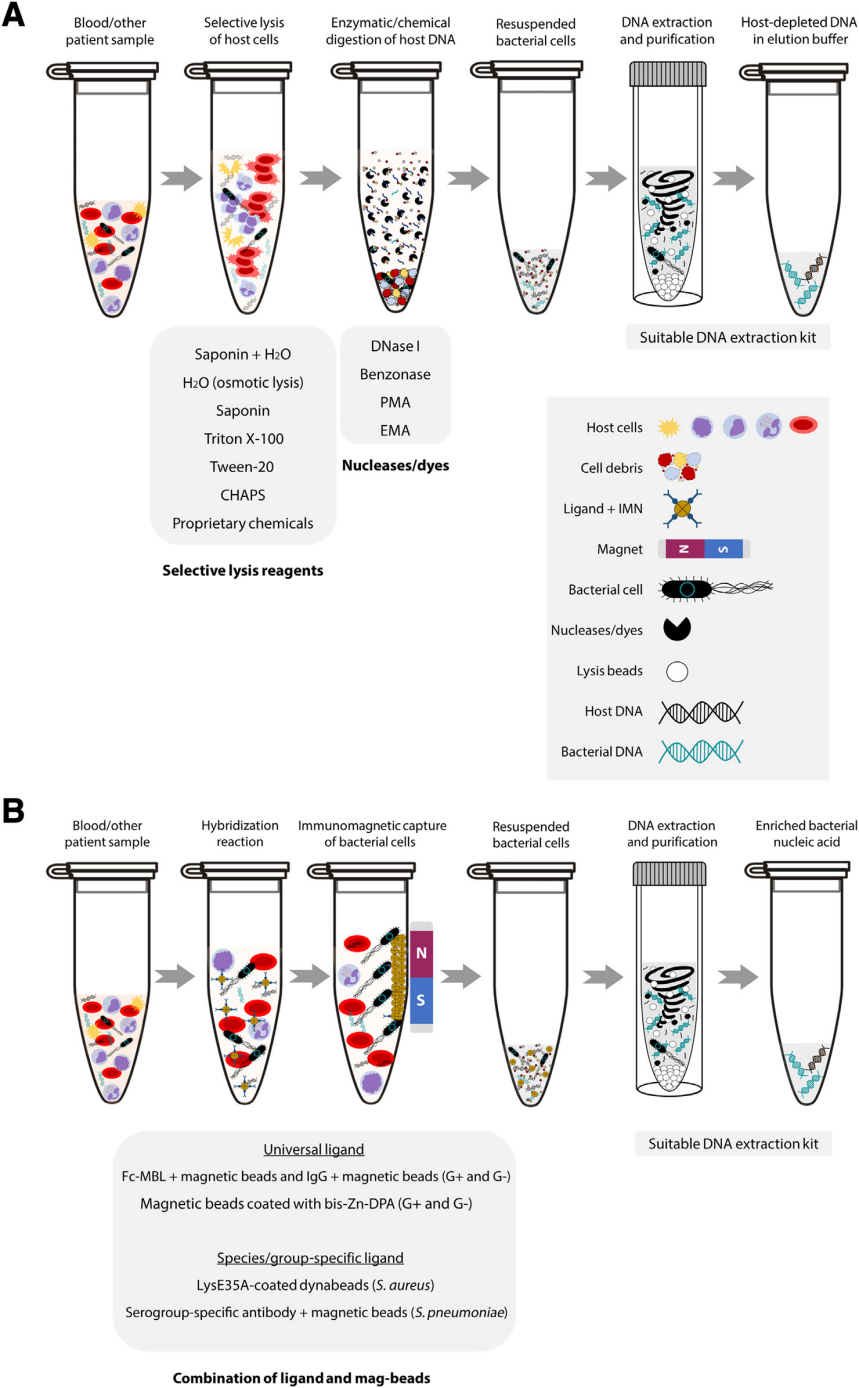
**A:** Major steps involved in chemical host depletion methods that aim to remove only the host DNA directly from patient samples. Host cells are selectively lysed using nonionic detergents, followed by the removal of host nucleic acid using DNA-reducing agents, enzymes, or dyes. **B:** Physicochemical approaches that selectively target and concentrate bacterial cells (enrichment) before DNA extraction is performed. CHAPS, 3-[(3-cholamidopropyl) dimethylammonio]-1-propanesulfonate; DPA, dipicolylamine; EMA, ethidium-monoazide; Fc-MBL, fragment crystallizable mannose binding lectin; IMN, immunomagnetic; PMA, propidium-monoazide; *S. aureus*, *Staphylococcus aureus*; *S. pneumoniae*, *Streptococcus pneumoniae*.

These approaches focus mostly on selective chemical lysis of host cells (eg, using nonionic detergents or proprietary chemicals), followed by host DNA degradation (eg, with DNase I, benzonase, or propidium-monoazide), total DNA extraction, and often total nucleic acid amplification to meet input requirements for sequencing (Figure 2A).^30^, ^31^, ^32^^,^^58^

Freshly collected samples, in comparison to frozen specimens, are most appropriate for these methods as they aim to selectively lyse host cells without compromising bacterial cell integrity. To deplete host, the utility of nonionic detergents, such as saponin, Triton X-100, Tween-20, or 3-[(3-cholamidopropyl) dimethylammonio]-1-propanesulfonate, has been described in several studies using samples including tissue, swabs, or body fluids, such as blood or cerebrospinal fluid.^33^^,^^65^ For example, one study in 2016 conducted by Hasan et al^33^ compared the host depletion efficiency of the aforementioned detergents with a commercial kit for real-time quantitative PCR– or sequencing-based bacterial identification. This study used spiked cerebrospinal fluid and nasopharyngeal aspirates and concluded that saponin and DNase I treatment performed better overall and removed >95% of human DNA without significantly affecting bacterial concentration compared with other kits or detergents, including Triton X-100, Tween-20, or 3-[(3-cholamidopropyl) dimethylammonio]-1-propanesulfonate.^33^

In 2018, Marotz et al^30^ compared a few commercially available kits [eg, QIAamp DNA Microbiome Kit (Qiagen, Hilden, Germany) and MolYsis Basic (Molzym GmbH & Co, Bremen, Germany)] with their own in-house protocol, lyPMA (osmotic lysis and treatment with propidium-monoazide). The in-house protocol uses water instead of detergents to selectively lyse host cells, and propidium-monoazide for DNA removal.^30^ Compared with the no host depletion controls (89.29% host DNA), the lyPMA protocol resulted in 8.53% host DNA in saliva samples, and the QIAamp DNA Microbiome and MolYsis Basic kit 29.17% and 62.88% host, respectively.^30^

Also, a 2019 study conducted by Charalampous et al^32^ described a depletion protocol that removed up to 99% of host nucleic acid from the 81 respiratory samples tested (ie, sputum, bronchoalveolar lavage, and endotracheal aspirates). This protocol uses saponin, followed by water, for selective lysis of host cells, heat liable salt active nuclease to digest the cell-free host nucleic acid, and ONT's Rapid PCR Barcoding Kit to amplify and barcode the low abundant bacterial DNA in the sample. This host depletion and amplification step significantly increased the clinical sensitivity (up to 100%) of the assay compared with culture, and the authors were able to identify bacterial species causing the lower respiratory tract infection and the associated antimicrobial resistance genes in <6 hours.^32^ In 2020, the same protocol was tested again with blood from healthy human volunteers and spiked bacterial species (*Yersinia pestis*, *Francisella tularensis*, and *Bacillus anthracis*), and up to 1 × 10^5^ times reduction of host DNA was observed.^57^

Another rapid protocol, termed as Sepsis@Quick, was evaluated in 2019 with 144 blood samples from patients.^6^ Although the primary goal of this workflow was to detect the bacterial species only with PCR, the authors described this protocol to be superior to blood culture in terms of clinical sensitivity (Sepsis@Quick versus blood culture: 57.63% versus 34.02%). This protocol uses a mammalian cell lysis buffer that contains Na_2_CO_3_ and Triton X-100, which, according to the authors, simultaneously lyses the host cells and degrades host nucleic acid without applying any DNAse or benzonase.^6^

More recently, some commercial host depletion kits, QIAamp DNA Microbiome (Qiagen), Molzym Ultra-Deep MicrobiomePrep (Molzym GmbH & Co), and Zymo HostZERO microbial DNA kit (Zymo Research, Irvine, CA), were tested on biopsy samples from infected foot tissue to evaluate their efficiency of host DNA removal.^31^ This study concluded that the top two performing kits reduced host DNA up to 32-fold (QIAamp DNA Microbiome) and 57-fold (Zymo HostZERO) compared with no depletion controls.

Although the analytical sensitivity of mNGS when combined with some of these approaches is low (between 10^3^ and 10^5^ CFUs/mL sample),^32^ and there are reports of potential bias (ie, bacterial cell lysis), its efficiency of host DNA removal was significant. Although some of these aforementioned studies did not use blood, they can be adapted for any sample type as long as they are freshly collected and contain intact bacterial cells. Therefore, it is worth testing and/or improving these workflows for the rapid molecular diagnosis of BSI.

### Pathogen Enrichment

As the name suggests, immunomagnetic approaches use antibodies or synthetic compounds with a high cell-binding affinity to separate bacteria from the sample following a successful hybridization with paramagnetic beads (Figure 2B). This technique has been used as a group/species-specific method (using narrow-spectrum antibodies) or as a universal approach (using Fc-mannose–binding lectin coated Fe_3_O_4_ or synthetic bis-zinc-dipicolylamine; both can hybridize with Gram-positive and Gram-negative bacteria) for bacterial enrichment in various sample types, including blood or urine.^34^, ^35^, ^36^ Several studies have also adapted this technique for use with physicochemical microfluidic devices.^37^^,^^38^

The effectiveness of this method in capturing bacteria can vary depending on sample type and bacterial species, and the capture efficiency can significantly decrease with complex samples with high viscosity. Additionally, the presence of hybridization inhibitors, such as lipids, proteins, and residual surface antigens from dead/lysed target cells, might further impede the process (Figure 2B).^66^ However, given the benefits, this technique can be explored further to increase capture efficiency and breadth of species it can target, thus enabling its use as a bacterial enrichment tool for blood samples.

## Physiochemical Methods Suitable for Purified Nucleic Acid

### Host Depletion

**Figure 3:**
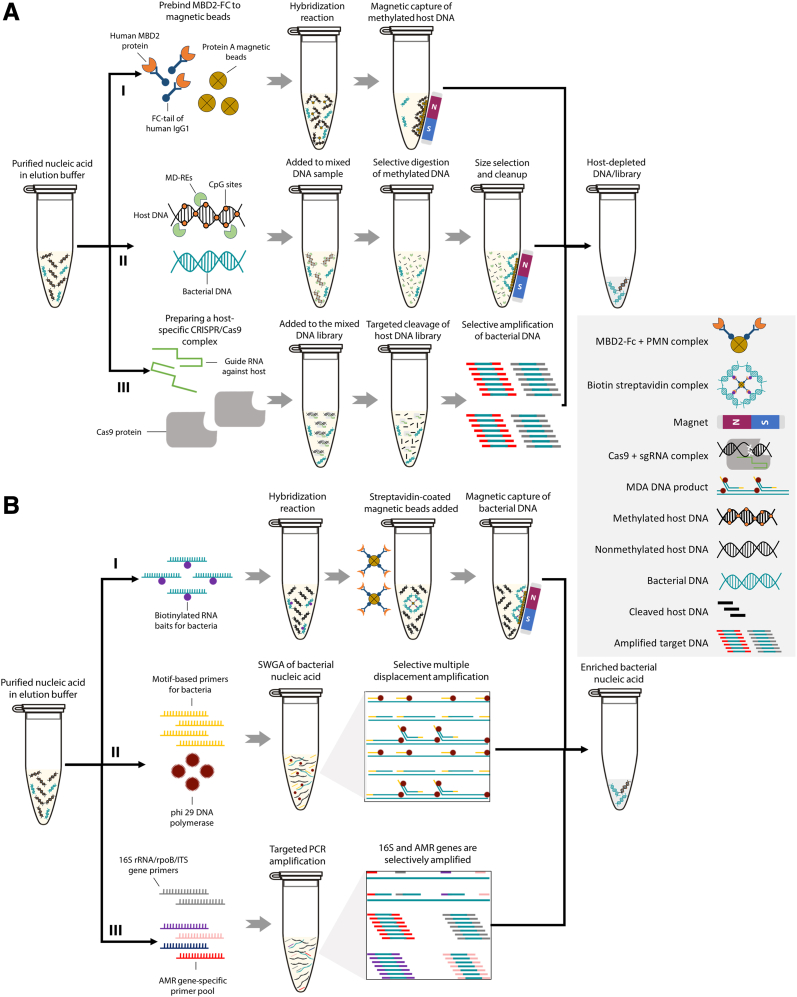
**A:** Major steps involved in a group of post-DNA extraction methods where unwanted host nucleic acid is removed using (I) human IgG1-bound methyl-CpG–binding domain protein 2 (MBD2) and magnetic beads (NEBNext Microbiome DNA Enrichment Kit), (II) methylation-dependent restriction endonucleases (MD-REs; eg, MsPJI, New England Biolabs), or (III) host-specific CRISPR/Cas9 and guide RNA (depletion of abundant sequences by hybridization). **B:** Major steps of three bacterial enrichment strategies that use purified DNA as a starting material. (I) Biotinylated RNA baits and magnetic beads are used to fish out targeted nucleic acid (eg, from a specific pathogen). (II) Bacterial motif-based primers and phi 29 DNA polymerase are used for selective whole-genome amplification (SWGA). (III) Amplification of genomic regions of bacteria is used to identify species (16S rRNA) and/or antimicrobial-resistant determinants with PCR. AMR, antimicrobial resistance; Fc, fragment crystallizable; ITS, internal transcribed spacer; MDA, multiple displacement amplification; PMN, protein A magnetic beads; sgRNA, single-guide RNA.

Methyl-CpG–binding domain protein 2 (MBD2)–based DNA capture is a methylation-dependent method to remove unwanted host from purified DNA samples.^31^^,^^39^^,^^67^ Because methylated CpG sites are almost exclusively present in human or other eukaryotic DNA, MBD2 can selectively bind and remove CpG-methylated host DNA in a sample with the help of magnetic beads [eg, NEBNext Microbiome DNA Enrichment Kit (New England Biolabs)] (Figure 3A, I).^68^ Although this approach was initially reported to deplete human DNA with high efficiency,^39^ many other studies have described its performance to be relatively low compared with other published or commercial host depletion methods.^31^^,^^58^^,^^69^ This is possibly attributable to binding efficiency of MBD2 and/or antibody complex or the methylation status of the host or microbial DNA.^68^ Methylated cytosines represent only 4% to 6% of all cytosines in a human genome, and the CpG sites that are unmethylated (10% to 40%) could be missed during depletion. Also, if nucleic acid is fragmented during DNA extraction, the capture efficiency of MBD2-based methods can significantly decrease because a large proportion of strands might lack methylated cytosines required for optimal separation.

Methylation-dependent restriction endonucleases are another choice of selective depletion/digestion of methylated host DNA. Like MBD2, restriction enzymes, such as MsPJI, FspEI, and LpnPI, have an affinity toward methylated cytosines (mostly present in host) and can selectively bind and make precise cuts between N12/N16 on the 3′ end of CpGs. To deplete host, some studies have used this selective enzymatic digestion step on extracted DNA from clinical samples and described 9- to 142-fold enrichment of pathogen compared with untreated controls (Figure 3A, II).^40^^,^^41^ This approach was also used in a later study; however, equal depletion of both host and parasite DNA was observed compared with the controls.^42^ Like MBD2, the efficiency of methylation-dependent restriction endonuclease–based approaches could be limited by similar factors. Only a limited number of studies have used this approach for infectious disease diagnosis; therefore, further investigation might be required to understand any potential detrimental effect of methylation-dependent restriction endonucleases on parasite or clinically relevant bacterial species.

Depletion of abundant sequences by hybridization (DASH) is a clustered regularly interspaced palindromic repeats (CRISPR)–associated protein 9 (Cas9) endonuclease-based method for depleting unwanted host nucleic acid (eg, rRNA transcripts), which relies on the ability of the CRISPR/Cas9 system to hybridize selectively to the host DNA in a mixed NGS library and cleave it with the help of host-specific guide RNA molecules (Figure 3A, III).^43^ Originally described by Gu et al,^43^ the DASH method was found to be highly effective, as up to fourfold enrichment of pathogenic sequences was reported following host depletion. A few studies, although not all aimed at infectious disease diagnosis, have also used DASH or similar approaches and described relatively high levels of host DNA reduction.^44^ Although DASH is robust and cost-effective compared with direct mNGS and the depletion efficiency of rRNA in cerebrospinal fluid samples has been reported to be between 81.4% and 88.2%, it has not been thoroughly examined using whole blood and/or rapid sequencing library kits/platforms for BSI diagnosis. Considering its utility, DASH or similar host depletion methods discussed in this section can be tested in conjunction with pre-DNA extraction methods or independently when working with purified nucleic acid.

### Pathogen Enrichment

Nucleic acid bait capture is a post-DNA extraction tool that can selectively enrich pathogen DNA in a sample. To separate bacterial DNA from a complex mixture, nucleic acid baits complementary to the target species are biotinylated (baits attached to a biotin molecule) through a chemical or enzymatic process (Figure 3B, I). These biotin-bound baits hybridize and form a complex with the target nucleic acid (eg, of the desired bacteria). With the help of streptavidin (binds to biotin with high affinity) coated magnetic beads, hybridized baits can later be separated from the unbound DNA and used for downstream assays, such as sequencing (Figure 3B, I).^45^ This sequence capture method has been reported for culture-independent enrichment of *Chlamydia* species, *Bacillus anthracis*, and *Mycoplasma amphoriforme* from vaginal swabs, animal carcass tissues, and nasopharyngeal swab samples, respectively, with a relatively high level of efficiency.^45^^,^^70^ Although these methods have shown promising results, a universal bait capture library that covers all the pathogenic species frequently observed in BSI has never been validated. Also, the analytical sensitivity of this approach is low and, therefore, requires low cycle threshold values, which typically correlate with higher bacterial loads.^45^ As a result, the utility of bait-capture is limited unless a more rapid, sensitive, and specific (minimal off-target binding) capture process and a universal bait library uniformly covering a large number of bacterial species (both intraspecies and interspecies targets) is developed and validated.

Rapid enrichment of bacterial DNA might also be possible with CRISPR/Cas9-mediated adapter ligation. The Cas9 Sequencing Kit (SQK-CS9109) offered by ONT can enrich regions of interest from purified nucleic acid samples. This kit works by making precise cuts and ligating sequencing adapters only to regions of interest with the help of Cas9/guide RNA–ribonucleoprotein. When the DNA library is loaded on a flow cell, only the adapter-ligated region of interest will be sequenced and as a result enriched. Although none of the studies reviewed here have used this technique for bacterial enrichment or as a rapid BSI diagnostic tool, it is theoretically feasible to enrich bacterial DNA directly from extracted patient samples, or chemically depleted/amplified DNA products with a comprehensive guide RNA library and some optimization.

Selective whole-genome amplification (SWGA) is a targeted DNA amplification method that uses a set of species-specific primers or motifs (8 to 12 bp) to selectively enrich bacterial DNA with the help of phi 29 polymerase-based isothermal amplification (multiple displacement amplification) (Figure 3B, II). Compared with conventional tiling PCR primers, commonly used for the enrichment of viral nucleic acid, SWGA primers are different in several ways. They have lower primer melting temperature (typically <30°C), which enables their isothermal use with multiple displacement amplification, and they target bacterial DNA motifs instead of conserved and complementary sequences used in tiling PCR.^46^ Because of this and the ability of multiple displacement amplification to produce high-molecular-weight products (approximately 10 kb), the total number of primers required to amplify a bacterial genome is significantly lower (between 20 and 40) than the tiling PCR-based approaches, which typically require >100 primers to cover a smaller viral genome (Figure 3B, II).^46^^,^^47^ In 2017, Clarke et al^48^ developed a motif-based primer design software (*https://www.github.com/eclarke/swga*) for SWGA-based amplification approaches and successfully used it to design and amplify *Wolbachia pipientis* and *Mycobacterium tuberculosis* genomic DNA from *Drosophila melanogaster* and human blood samples, respectively. Recently, other studies have also effectively implemented this approach for detecting *Coxiella burnetii* from the environment and *Neisseria meningitidis* from clinical samples.^47^^,^^71^ Although SWGA is rapid, robust, and sensitive for single species identification, its utility as a general BSI diagnosis tool has not been validated. Although designing a large set of motifs for detecting bloodstream infections caused by a plethora of bacterial species would be challenging, it is worth investigating the potential of SWGA as a part of rapid BSI diagnosis workflow.

Targeted amplicon sequencing (TAS) is another selective nucleic acid enrichment technique. Unlike SWGA, TAS is mostly applied to enrich specific genomic regions of a pathogen to detect species (eg, the universal 16S rRNA gene for bacteria or internal transcribed spacer regions for fungi) or identify AMR determinants from a mixed DNA sample.^49^^,^^72^ This is done primarily by PCR amplification of regions of interest with target-specific oligonucleotides and then sequencing them to derive valuable information (Figure 3B, III). Although this approach can detect a limited number of AMR genes, it is far cheaper than mNGS-based approaches and has been described to identify bacterial species and a few AMR determinants with different sample types. For example, in 2023, Zhang et al^49^ clinically evaluated one such approach, wherein 16S rRNA + *rpoB* and internal transcribed spacer primers were used to identify bacterial or fungal species, respectively. Multiplex PCR panels that can detect both AMR and bacterial species using TAS have been tested for rapid clinical diagnosis with various clinical samples, however, with smaller collection of primers or targets.^50^^,^^73^ More comprehensive AMR panels capable of sequencing hundreds of AMR genes at a time are available for various sequencing platforms, including Illumina and Ion Torrent GeneStudio (eg, AmpliSeq or DARTE-QM^51^), but none of them have been used for rapid clinical management of BSI. Although TAS approaches are not as comprehensive as mNGS and might miss novel AMR determinants, their high analytical sensitivity and robustness make them a suitable candidate for situations where only a limited amount of information (eg, species and/or AMR) is sufficient to make lifesaving clinical decisions.

## Selective Sequencing to Deplete Host or Enrich Pathogen

**Figure 4:**
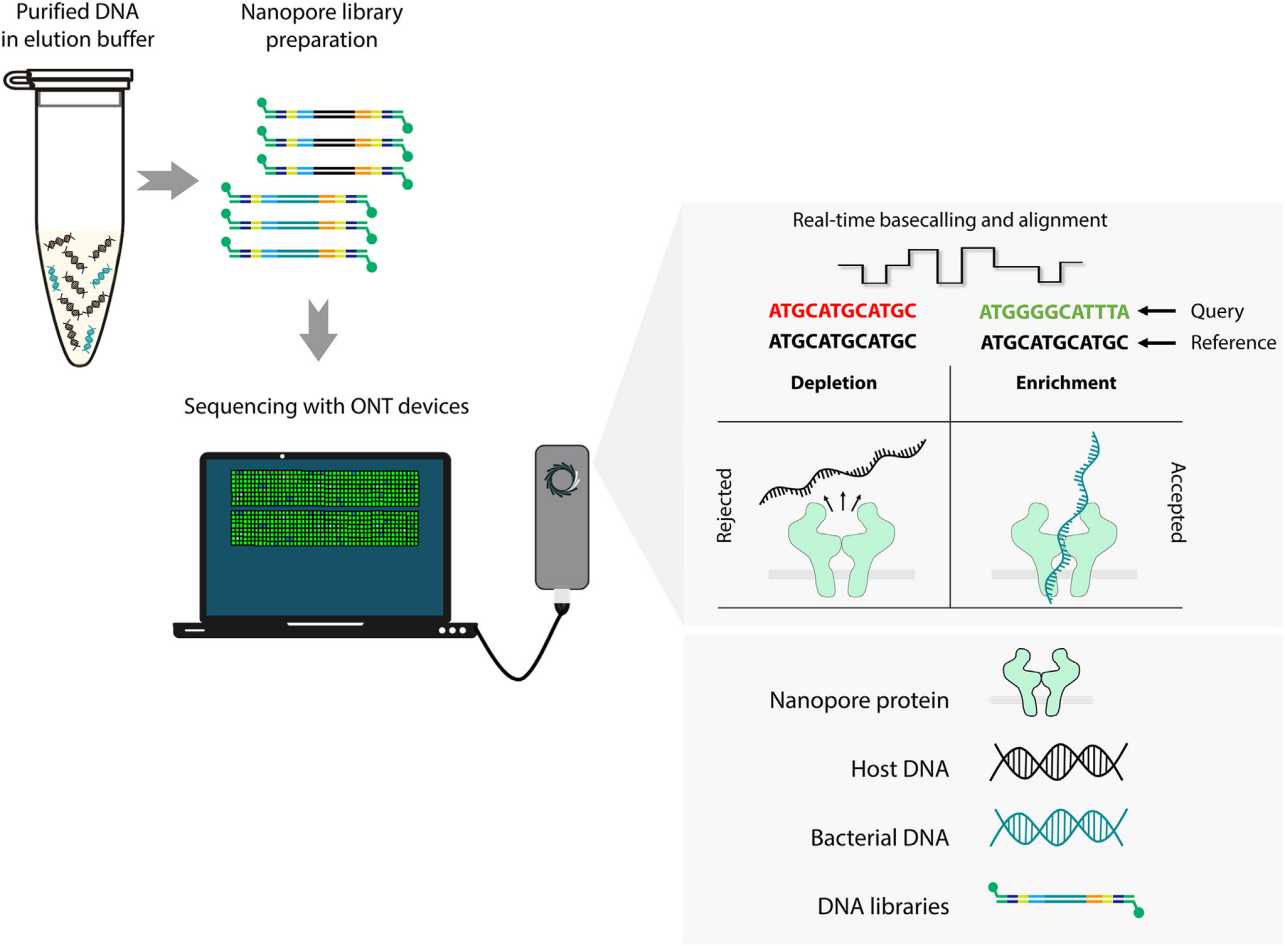
Selective sequencing, a software-based approach to remove unwanted host DNA (depletion) or enrich desired bacterial nucleic acid (enrichment) in real time using Oxford Nanopore Technologies (ONT) devices. With this approach, live signals streamed during a sequencing run can be mapped onto a reference sequence(s), and enrichment or depletion can be performed by ejecting unwanted DNA molecules by reversing the voltage across the protein nanopores.

Selective sequencing (alias adaptive sampling) is a depletion/enrichment approach unique to the ONT sequencing platforms, where unwanted DNA strands can be selectively rejected in real time during a sequencing run.^52^ ONT sequencing devices generate data by measuring real-time changes in electrical current while DNA molecules pass through the protein nanopores. These devices can stream data in real time and simultaneously control the movement of DNA molecules by manipulating the voltage across the pores on individual channels. The software underpinning this capacity, originally termed as Read Until, has expanded and improved over time (Read Until,^52^ RUBRIC,^53^ UNCALLED,^54^ Readfish,^55^ and ReadBouncer^56^), and is used to remove unwanted DNA by depleting the host or enriching the bacterial species during nanopore sequencing (Figure 4). The efficiency of these approaches primarily depends on the length of DNA strands (fragmented DNA = lower efficiency), computational capability [fewer central processing unit/graphics processing unit (CPU/GPU) cores = lower efficiency], translocation speed of the DNA through the pores (higher speed = lower efficiency), and the size of the reference genome/database (larger genome/database = lower efficiency) used for enrichment or depletion.

This software-based approach does not require any additional sample preparation steps, like other post-DNA extraction methods (saving time and cost), and can enrich least abundant species up to 13.87-fold with a high-performance GPU if length of the DNA library is optimal (>10 kbp).^74^ Some implementations of selective sequencing have reported significant removal of unwanted DNA molecules even with gigabase-sized reference genomes. However, until recently, a limitation of the approach has been the requirement to implement the available selective sequencing software tools as a third-party application (command line based), requiring some expertise.^54^^,^^55^ To address this limitation, ONT recently integrated the CPU- or GPU-based Readfish software into its MinKNOW control application, expanding its accessibility for more widespread users (Figure 4).^74^ Taking this into consideration, it is also worth exploring the potential of adaptive sampling as a standalone method or together with pre-DNA and/or post-DNA extraction host depletion techniques.

## Concluding Remarks and Future Perspectives

Rapid identification of the pathogen and AMR profiles in BSI is crucial as it can enable timely and targeted antimicrobial therapy and reduce detrimental outcomes by minimizing the progression and severity of infections. It may also help to reduce the use of broad-spectrum antibiotics, and thereby contribute to preserving their effectiveness and minimizing the increase of newer antibiotic-resistant strains.

Rapid sequencing, especially in combination with selective host depletion/bacterial enrichment approaches, has the potential to accelerate microbiological diagnosis of BSI and inform targeted antibiotic prescribing. Although most of the techniques discussed in this review possess their individual strengths, none are comprehensive and might require further improvements to enable their use for the rapid detection of species and AMR determinants. For example, physical approaches, such as filtration and centrifugation, are rapid and cost-effective; however, recovery rate of bacteria and/or depletion efficiency as a standalone method have been described to be less than optimal for direct mNGS, especially for samples with low bacterial abundance, such as blood. Numerous sequencing-based depletion/enrichment workflows have combined both physical and chemical approaches (physicochemical) and described significant improvements.^31^^,^^32^^,^^45^ However, some of these methods, when tested directly on blood or other body fluids, showed reduced pathogen recovery^32^^,^^57^ or less than optimal host depletion efficiency,^31^ or there was no emphasis placed on turnaround time, as sequencing was performed on relatively slower mNGS workflows.^43^ Some of the targeted approaches have been reported to be more sensitive, cost-effective, and rapid but developed and tested as syndromic panels to detect only the bacterial species and/or a small collection of AMR determinants.^48^^,^^49^

It is also important to recognize that most of these physiochemical approaches (before/after extraction) have never been amalgamated and/or tested for rapid BSI diagnosis. Consequently, these approaches were categorized into a few distinguishable groups to highlight the fact that these individual methods, although not comprehensive, could be combined to harness their collective strengths.

For low abundant samples, like blood, steps (depletion/enrichment) leading to the end of DNA extraction are delicate and must reliably recover most bacteria. Although higher bacterial proportion following depletion/enrichment is desirable for mNGS, it is also important to ensure that pathogen DNA is not lost at this stage. As there are options to amplify total DNA (eg, PCR or multiple displacement amplification with random primers) following successful retention of pathogens' nucleic acid, additional steps (selective enrichment or depletion) can be taken using extracted DNA to further improve host/bacterial proportions once the risk of losing the most desired information is lower. Targeted DNA amplification (eg, SWGA or TAS) could be another suitable alternative to unbiased amplification, as this will further enrich bacterial DNA from the sample if the initial host depletion step does not yield desired proportions. A comprehensive panel of primers (eg, motif based for SWGA or conventional PCR for amplicon) might help increase the detection breath; however, the choice of oligos as well as targets needs to be thoroughly investigated and validated for this purpose. Because some of these additional steps might increase overall hands-on time, cost, and the chances of cross-contamination, selective sequencing can provide another viable solution. Other than a capable GPU, no extra reagents or sample processing is required with this approach, although this option is limited only to ONT devices and requires a fairly high-molecular-weight DNA library for optimal yield, and efficient depletion/enrichment.^74^

Although the future of rapid sequencing-based methods looks bright, it is also important to realize that the presence or absence of AMR genes does not necessarily mean a bacterium is phenotypically resistant or, more importantly, susceptible to a particular antibiotic. A new system/workflow has to demonstrate ≥90% categorical agreement, ≤3% major error (false positivity; might influence AMR), and ≤1.5% very major error (false negativity; may lead to treatment failure) for it to be accepted in a clinical setup in many countries.^75^ Currently, sequencing-based AMR identification methods [eg, CARD (*https://card.mcmaster.ca*) and ResFinder (*http://genepi.food.dtu.dk/resfinder*)] may exhibit between 25% and 42% major error, leading to possible overtreatment, but relatively lower risk of treatment failure, with 1.17% to 4.42% very major error.^76^ To improve performance, some studies are using transcriptomic or proteomic approaches and/or machine learning models for more reliable prediction of antimicrobial resistance.^77^ However, more efforts are required in this area to make sequencing results more accurate and comprehensible to nonspecialists (eg, laboratory personnel and health care practitioners).

Taken together, this review describes a range of host depletion/pathogen enrichment approaches and highlights some of their limitations when used as a stand-alone method for preparing samples for rapid sequencing-based BSI diagnosis. Although a few of them work exceedingly well in some sample types/syndromes, it is crucial to understand critical steps that can be modified or replaced to overcome their limitations when applied to blood or any sample with high host background and low abundance of bacteria. Merging two or more approaches into a single workflow can be considered if they are complementary (eg, before and after DNA extraction) and do not significantly increase turnaround time, cost, or complexity. Ultimately, further efforts are necessary to test and validate these current and emerging approaches to enable the use of sequencing for the rapid diagnosis of bloodstream infection.

## Disclosure Statement

None declared.
